# Supplementary material for: Xpert MTB/RIF Ultra versus Xpert MTB/RIF for diagnosis of tuberculous pleural effusion: A systematic review and comparative meta-analysis
Source: PLoS One. 2022 Jul 11;17(7):e0268483. doi: 10.1371/journal.pone.0268483 (PMC9273090; doi:10.1371/journal.pone.0268483)
Supplement: S1 Table — (PDF) [file pone.0268483.s002.pdf]

S1 Table. Characteristics of studies included in data synthesis.

Author, year	Country	Prospective	Case Control design	Inclusion criteria	Exclusion criteria	Sample size	Age in years*	HIV sero-positive patients	Controls	Index test(s)	Reference standard
Armand, 2011	France	No	Yes	Cryopreserved, mycobacterial culture positive and negative pleural fluid specimens from inpatients	None	8	NS	NS	NS	Xpert	Culture
Causse, 2011	Spain	Yes	No	NS	NS	34	Median 45	NS	NS	Xpert	Culture
Friedrich, 2011	South Africa	Yes	No	Undiagnosed pleural effusion with high suspicion of TB	NS	25	NS	NS	Other pleural infections	Xpert	Culture, Composite
Hanif, 2011	Kuwait	Yes	No	NS	NS	11	NS	NS	NS	Xpert	Culture
Malbruny, 2011	France	Yes	No	Clinical suspicion of TB	NS	12	NS	NS	NS	Xpert	Culture
Vadwai, 2011	India	Yes	No	Clinical suspicion of TB	ATT in past 60 days, insufficient clinical details or specimen volume, death or loss to follow up, NTM growth on culture	29	NS	NS	NS	Xpert	Culture
Zeka, 2011	Turkey	No	No	Cryopreserved specimens from patients with clinical suspicion of TB	History of ATT intake	56	NS	NS	NS	Xpert	Culture, Composite
Al-Ateah, 2012	Saudi Arabia	Yes	No	Clinical suspicion of TB	NS	13	NS	None	NS	Xpert	Culture
Moure, 2012	Spain	No	Yes	Smear negative cryopreserved fluid samples	NS	31	NS	NS	NS	Xpert	Culture

Safianowska, 2012	Poland	Yes	No	NS	NS	32	NS	NS	NS	Xpert	Culture
Tortoli, 2012	Italy	No	No	Samples from patients with clinical suspicion of TB	NS	330	NS, 11.5% children	NS	NS	Xpert	Culture, Composite
Christopher, 2013	India	Yes	No	Clinical suspicion of TB	NS	91	Median 46 (33-57)	NS	NS	Xpert	Composite
Porcel, 2013	Spain	Yes	Yes	Cryopreserved samples from patients with pleural effusion diagnosed as TB or non-TB	NS	67	Mean 33 (TB) and 67 (non-TB)	NS	Malignancy, parapneumonic, others	Xpert	Culture, Composite
Zmak, 2013	Croatia	Yes	No	Clinical suspicion of TB	NS	42	NS	NS	NS	Xpert	Culture
Lusiba, 2014	Uganda	Yes	No	Adults (>=18 years age) with exudative pleural effusion suspected to be TB and with fluid volume amenable to pleural biopsy	Contraindication to pleural biopsy, history of ATT intake	116	Mean 34±13	52/116	Malignancy, chronic inflammation, undiagnosed	Xpert	Composite
Meldau, 2014	South Africa	Yes	No	Clinical suspicion of TB	ATT for >2 days, incomplete data	88	Median 39 (TB) and 61 (non-TB)	11/65	Malignancy, parapneumonic, others	Xpert	Composite
Ozkutuk, 2014	Turkey	Yes	No	Clinical samples for which mycobacterial culture and GeneXpert were both performed	Contaminated cultures	232	NS	NS	NS	Xpert	Culture
Scott, 2014	South Africa	Yes	No	Samples received in Mycobacteriology laboratory	NS	482	NS	NS	NS	Xpert	Culture
Sharma, 2014	India	Yes	No	Clinical suspicion of TB	ATT for >=2 weeks, insufficient sample, inconclusive test results	364	NS	NS	NS	Xpert	Culture

Trajman, 2014	Brazil	Yes	No	Cryopreserved samples from inpatients needing diagnostic thoracentesis	Insufficient pleural fluid volume, coagulopathy, final diagnosis not confirmed	85	Median 50 (40-57)	5/61	Malignancy, liver cirrhosis, cardiac failure, Meigs' syndrome	Xpert	Composite
Coleman, 2015	Malawi	Yes	Yes	HIV seropositive adults with new pleural effusion	Treatment for TB or Kaposi's sarcoma	50	Mean 32	All	Kaposi's sarcoma	Xpert	Culture
Du, 2015	China	Yes	No	Adult inpatients requiring diagnostic thoracentesis	Sputum smear positive for acid-fast bacilli, absence of a paired pleural fluid/ pleural biopsy sample, inconclusive test results	126	39±13	5/126	NS	Xpert	Culture
Kim, 2015	Korea	No	No	Clinical suspicion of TB	NS	32	NS	NS	Malignancy, empyema, transudates	Xpert	Composite
Kim, 2015	Korea	No	No	Samples from inpatients with clinical suspicion of TB	Patients on ATT, microbiological data not available	388	NS	NS	NS	Xpert	Culture, Composite
Rufai, 2015	India	Yes	No	Clinical suspicion of TB	NS	161	42±19 for men, 39±19 for women	None	NS	Xpert	Culture
Mazzola, 2016	Italy	No	No	NS	Contaminated culture, clinical data not available	714	NS	NS	NS	Xpert	Culture
Nataraj, 2016	India	Yes	No	Clinical suspicion of TB	Inadequate specimen volume	167	NS	NS	NS	Xpert	Culture
Penata, 2016	Colombia	Ambispective	No	Clinical suspicion of TB	Inadequate clinical information, culture contamination	48	NS	NS	NS	Xpert	Culture
Suzana, 2016	India	Yes	No	Clinical suspicion of TB	NTM growth on culture	53	NS	NS	NS	Xpert	Culture

Yuan, 2016	China	No	No	Inpatients with clinical suspicion of TB	Smear microscopy or culture reports unavailable	63	NS	NS	NS	Xpert	Composite
Che, 2017	China	Yes	No	Inpatients >=18 years with clinical suspicion of TB	Incomplete clinical or outcome data	78	Median 44 (18-83)	1/78	NS	Xpert	Composite
Jing, 2017	China	Yes	No	Clinical suspicion of TB	NS	124	NS	NS	NS	Xpert	Culture
Li, 2017	China	Yes	No	Clinical suspicion of TB	Inconclusive test results, culture contamination	221	Mean 48±10	NS	NS	Xpert	Culture
Pandey, 2017	Australia	Yes	No	Samples from patients with clinical suspicion of TB	Inconclusive test results	22	NS	NS	NS	Xpert	Culture
Saeed, 2017	Pakistan	Yes	No	Samples from patients with clinical suspicion of TB	Contaminated samples, previously diagnosed TB cases, patients on ATT	158	NS	NS	NS	Xpert	Culture
Bankar, 2018	India	Yes	No	Samples from patients with clinical suspicion of TB	Inconclusive test results, culture contamination	304	NS	NS	NS	Xpert	Culture
Khan, 2018	Pakistan	Yes	No	Clinical suspicion of TB	NS	259	NS	NS	NS	Xpert	Culture
Perez-Risco, 2018	Spain	No	No	Smear negative cryopreserved fluid samples	NS	24	NS	NS	NS	Ultra	Culture
Prakash, 2018	India	Yes	No	Clinical suspicion of TB	Culture contaminated or not sent	193	NS	NS	NS	Xpert	Culture
Rakotoarivelo, 2018	Madagascar	Yes	No	Clinical suspicion of TB	NS	43	NS	NS	NS	Xpert	Culture, Composite
Sharma, 2018	India	Yes	No	Clinical suspicion of TB	Patients on ATT, incomplete data, HIV seropositivity, low sample volume, test results inconclusive	78	Mean 39	None	Lung and other malignancies, nephrotic syndrome	Xpert	Composite

Allahyartorkaman, 2019	Iran	Yes	No	Samples from patients with clinical suspicion of TB	NS	168	NS	NS	NS	Xpert	Culture
El-Din, 2019	Egypt	Yes	No	Clinical suspicion of TB	NS	58	Mean 33±14	NS	NS	Xpert	Composite
Kumari, 2019	India	Yes	No	Cryopreserved samples from patients with clinical suspicion of TB	Patients receiving ATT for >7 days, past history of TB, no final diagnosis	81	15-70	NS	Parapneumonic, liver/renal disease, vasculitis, heart failure, malignancy	Xpert	Composite
Liang, 2019	China	No	No	Clinical suspicion of TB, availability of pleural fluid test results	Patients receiving ATT, invalid test results	219	NS	NS	Malignancy, parapneumonic	Xpert	Composite
Luo, 2019	China	Yes	No	Clinical suspicion of TB	Age <18 years, indeterminate IGRA result, patients on ATT, lost to follow up, no final diagnosis	428	NS	NS	NS	Xpert	Composite
Mechal, 2019	Morocco	No	No	Samples from patients with clinical suspicion of TB	NS	65	NS	NS	NS	Xpert	Culture
Meldau, 2019	South Africa	Yes	No	Clinical suspicion of TB	NS	133	Median 39 (28-57)	17/120	Malignancy, parapneumonic, others	Xpert, Ultra	Composite
Tadesse, 2019	Ethiopia	Yes	No	Clinical suspicion of TB	Uncertain diagnosis, incomplete medical records	107	NS	NS	NS	Xpert	Culture, Composite
Tahseen, 2019	Pakistan	Yes	No	Clinical suspicion of TB	History of prior ATT, previously established TB diagnosis	279	Median 26 (21-37)	NS	NS	Xpert	Culture
Wang, 2019	China	Yes	No	Cryopreserved fluid samples from patients with clinical suspicion of TB	ATT in past 6 months, inconclusive test results	131	Median 37 (15-89)	None	NS	Xpert, Ultra	Culture, Composite

Wu, 2019	China	Yes	No	Clinical suspicion of TB, age >=16 years, HIV seronegative	Inconclusive test results, culture contamination, no definite diagnosis	129	NS	None	NS	Xpert, Ultra	Culture, Composite
Zhou, 2019	China	Yes	No	Inpatients with clinical suspicion of TB	Incomplete clinical/microbiologic al data, insufficient specimen	11	NS	NS	NS	Xpert	Composite
Abdelfattah, 2020	Egypt	Yes	No	Clinical suspicion of TB, age 18-80 years	Patients with malignancy or other underlying respiratory disease, HIV seropositivity, use of immunosuppressants	6	NS	None	NS	Xpert	Culture
Chen, 2020	China	No	No	Clinical suspicion of TB	Culture results not available	7	NS	NS	NS	Xpert	Composite
Dahiya, 2020	India	Yes	No	Clinical suspicion of TB	Drug-resistant TB, patients on ATT, diabetes, pregnancy, HIV seropositivity	42	NS	None	NS	Xpert	Composite
Han, 2020	China	Yes	No	Inpatients with exudative pleural effusion and clinical suspicion of TB	Age <18 years, HIV seropositivity, no definite diagnosis	265	Mean 41±18 (TB) and 56±14 (non-TB)	None	Malignancy, parapneumonic, others	Xpert	Culture
Hoel, 2020	Norway	Yes	No	Cryopreserved specimens from patients with clinical suspicion of TB	Pleural fluids with assumed low probability of TB	18	NS	NS	NS	Ultra	Culture
Li, 2020	China	No	No	Inpatients simultaneously tested with Xpert, smear microscopy for acid-fast bacilli, and IGRA	NS	82	NS	NS	NS	Xpert	Composite
Sasikumar, 2020	India	Yes	No	Clinical suspicion of TB	Incomplete data	41	NS	NS	NS	Xpert	Composite

Sumalani, 2020	Pakistan	Yes	No	Lymphocytic exudative pleural effusion, age >=18 years, unable to expectorate sputum	HIV seropositivity, lung parenchymal abnormalities on imaging, ATT for >1 week	148	Mean 37±17 (TB) and 39±16 (non-TB)	None	NS	Xpert	Composite
Wang, 2020	China	Yes	No	Clinical suspicion of TB; cryopreserved fluid used for Xpert Ultra assay	ATT in past 6 months, contaminated culture, inconclusive test results, lost to follow up	292	Median 45 (18-89)	None	Malignancy, parapneumonic, others	Xpert, Ultra	Culture, Composite
Yang, 2020	China	Yes	No	Clinical suspicion of TB, age >18 years	No definite diagnosis, test results not available	286	Median 46 (27-60)	NS	TB, malignancy, and pneumonia	Xpert	Composite
Yu, 2020	China	Yes	No	Samples from patients with clinical suspicion of TB	NS	80	NS	NS	NS	Xpert	Composite
Elbrolosy, 2021	Egypt	No	No	Samples from patients with clinical suspicion of TB	NS	24	NS	NS	NS	Xpert	Culture
Gao, 2021	China	Yes	No	Unexplained exudative pleural effusion, age 15-80 years	Not fit for video-assisted thoracoscopic pleural biopsy	61	Mean 42±18 (TB) and 62±12 (non-TB)	None	Malignancy, chronic inflammation	Xpert, Ultra	Culture, Composite
Guo, 2021	China	No	No	TB, malignancy, pneumonia, and other causes of pleural effusion	Uncertain diagnosis, missing tests	127	Mean 48±22	1/117	TB, malignancy, and pneumonia	Xpert	Composite
Kim, 2021	Korea	No	No	Samples from patients with clinical suspicion of TB	NS	111	NS	NS	NS	Xpert	Culture
Kobra, 2021	Bangladesh	Yes	No	Clinical suspicion of TB	Previous diagnosis of TB or taking ATT	55	10-75	NS	NS	Xpert	Culture
Koumeka, 2021	Morocco	Yes	Yes	Inpatients undergoing pleural biopsy	Pleural histopathology results unavailable	45	Mean 36±18	None	Malignancy, others, nonspecific	Xpert	Composite

Lopez-Roa, 2021	Spain	No	No	Samples from patients with clinical suspicion of TB	NS	118	NS	NS	NS	Ultra	Culture
Mekkaoui, 2021	Belgium	No	No	Samples from patients with clinical suspicion of TB	NS	77	NS	NS	NS	Ultra	Culture
Penata-Bedoya, 2021	Colombia	No	No	Samples from patients with clinical suspicion of TB	Incomplete medical records, technical issues, culture contamination	93	NS	NS	NS	Ultra	Culture
Sun, 2021	China	Yes	No	Inpatients with clinical suspicion of TB, age >=18 years	Inability to perform pleural biopsy, HIV seropositivity	234	Median 31 (TB) and 45 (non-TB)	None	Malignancy, non-tubercular mycobacterial infection	Xpert	Composite

ATT Anti-tuberculous treatment, HIV Human immunodeficiency virus, IGRA Interferon-gamma release assay, NTM Non-tuberculous mycobacteria, NS Not specified, TB Tuberculosis

* Figures are mean ± standard deviation, or median (interquartile range), as appropriate
