# Supplementary material for: Xpert MTB/RIF Ultra versus Xpert MTB/RIF for diagnosis of tuberculous pleural effusion: A systematic review and comparative meta-analysis
Source: PLoS One. 2022 Jul 11;17(7):e0268483. doi: 10.1371/journal.pone.0268483 (PMC9273090; doi:10.1371/journal.pone.0268483)
Supplement: S2 Table — (PDF) [file pone.0268483.s003.pdf]

S2 Table. Diagnostic accuracy estimates from included studies.

Author, year	Index test	Reference standard	Sensitivity (95% CI)	Specificity (95% CI)	Positive likelihood ratio (95% CI)	Negative likelihood ratio (95% CI)	Diagnostic odds ratio (95% CI)
Armand, 2011	Xpert	Culture	0.43 (0.10-0.82)	1.00 (0.03-1.00)	1.33 (0.23-7.74)	0.83 (0.31-2.24)	1.60 (0.10-24.70)
Causse, 2011	Xpert	Culture	1.00 (0.40-1.00)	1.00 (0.88-1.00)	26.67 (3.75-189.7)	0.17 (0.03-1.03)	155.00 (8.29-2897)
Friedrich, 2011	Xpert	Culture	0.26 (0.09-0.51)	1.00 (0.54-1.00)	2.29 (0.32-16.13)	0.82 (0.56-1.19)	2.80 (0.28-27.91)
		Composite	0.25 (0.09-0.49)	1.00 (0.48-1.00)	1.91 (0.27-13.27)	0.85 (0.57-1.26)	2.25 (0.22-22.79)
Hanif, 2011	Xpert	Culture	1.00 (0.29-1.00)	1.00 (0.63-1.00)	8.00 (1.18-54.04)	0.22 (0.04-1.30)	36.00 (1.77-731.6)
Malbruny, 2011	Xpert	Culture	0.00 (0.00-0.84)	1.00 (0.69-1.00)	3.00 (0.24-37.67)	0.82 (0.45-1.48)	3.67 (0.17-77.55)
Vadwai, 2011	Xpert	Culture	0.50 (0.19-0.81)	1.00 (0.82-1.00)	10.50 (1.43-77.17)	0.53 (0.30-0.93)	20.00 (1.99-200.5)
Zeka, 2011	Xpert	Culture	0.00 (0.00-0.60)	1.00 (0.93-1.00)	9.00 (0.64-126.2)	0.85 (0.59-1.22)	10.60 (0.57-196.5)
		Composite	0.00 (0.00-0.37)	1.00 (0.93-1.00)	5.00 (0.34-73.46)	0.92 (0.74-1.13)	5.44 (0.31-95.21)
Al-Ateah, 2012	Xpert	Culture	1.00 (0.29-1.00)	1.00 (0.69-1.00)	9.60 (1.40-65.94)	0.22 (0.04-1.27)	44.00 (2.19-882.7)
Moure, 2012	Xpert	Culture	0.27 (0.12-0.48)	1.00 (0.48-1.00)	2.00 (0.30-13.46)	0.83 (0.57-1.22)	2.40 (0.25-23.24)
Safianowska, 2012	Xpert	Culture	0.00 (0.00-0.84)	1.00 (0.88-1.00)	8.00 (0.61-104.5)	0.77 (0.44-1.37)	10.33 (0.51-210.6)
Tortoli, 2012	Xpert	Culture	0.33 (0.12-0.62)	0.99 (0.97-1.00)	35.00 (9.22-132.9)	0.67 (0.47-0.96)	52.00 (10.89-248)
		Composite	0.44 (0.22-0.69)	1.00 (0.99-1.00)	141.30 (18.8-1060)	0.55 (0.37-0.82)	256.09 (29.78-2202)
Christopher, 2013	Xpert	Composite	0.13 (0.04-0.31)	1.00 (0.94-1.00)	9.84 (1.20-80.74)	0.86 (0.74-1.00)	11.48 (1.28-103.0)
Porcel, 2013	Xpert	Culture	0.40 (0.05-0.85)	0.95 (0.86-0.99)	8.13 (1.74-37.92)	0.63 (0.31-1.29)	12.89 (1.53-108.7)
		Composite	0.15 (0.05-0.32)	1.00 (0.90-1.00)	6.17 (0.78-48.68)	0.85 (0.73-1.00)	7.24 (0.82-63.64)
Zmak, 2013	Xpert	Culture	0.00 (0.00-0.98)	1.00 (0.91-1.00)	14.33 (1.16-176.8)	0.68 (0.31-1.52)	21.00 (0.93-472.6)
Lusiba, 2014	Xpert	Composite	0.29 (0.20-0.39)	0.97 (0.82-1.00)	8.33 (1.18-58.81)	0.74 (0.64-0.86)	11.29 (1.46-87.53)
Meldau, 2014	Xpert	Composite	0.23 (0.11-0.38)	0.98 (0.89-1.00)	10.80 (1.43-81.65)	0.79 (0.67-0.94)	13.65 (1.65-113.1)
Ozkutuk, 2014	Xpert	Culture	0.40 (0.05-0.85)	1.00 (0.98-1.00)	98.14 (11.61-829)	0.57 (0.30-1.09)	171.00 (14.47-2020)

Scott, 2014	Xpert	Culture	0.47 (0.38-0.56)	0.94 (0.91-0.96)	8.02 (5.09-12.64)	0.56 (0.47-0.66)	14.30 (8.14-25.13)
Sharma, 2014	Xpert	Culture	0.41 (0.30-0.51)	0.97 (0.94-0.99)	13.88 (6.71-28.69)	0.61 (0.52-0.73)	22.70 (10.01-51.4)
Trajman, 2014	Xpert	Composite	0.03 (0.00-0.12)	1.00 (0.87-1.00)	1.38 (0.15-12.66)	0.99 (0.90-1.08)	1.40 (0.14-14.05)
Coleman, 2015	Xpert	Culture	0.69 (0.39-0.91)	1.00 (0.91-1.00)	26.00 (3.63-186.0)	0.34 (0.17-0.70)	76.00 (7.95-726.3)
Du, 2015	Xpert	Culture	0.44 (0.30-0.58)	0.99 (0.92-1.00)	30.98 (4.32-222.0)	0.57 (0.45-0.72)	54.19 (7.01-418.7)
Kim, 2015	Xpert	Composite	0.00 (0.00-0.46)	1.00 (0.87-1.00)	3.50 (0.25-49.94)	0.91 (0.69-1.19)	3.86 (0.21-69.67)
Kim, 2015	Xpert	Culture<br>Composite	0.15 (0.02-0.45)<br>0.10 (0.03-0.22)	0.99 (0.98-1.00)<br>1.00 (0.99-1.00)	19.23 (3.51-105.4)<br>40.12 (4.93-326.5)	0.85 (0.68-1.08)<br>0.88 (0.80-0.98)	22.55 (3.42-148.8)<br>45.33 (5.34-385.2)
Rufai, 2015	Xpert	Culture	0.55 (0.39-0.70)	1.00 (0.97-1.00)	66.00 (9.20-473.4)	0.46 (0.33-0.63)	144.00 (18.43-1125)
Mazzola, 2016	Xpert	Culture	0.38 (0.18-0.62)	1.00 (0.99-1.00)	271.96 (35.9-2057)	0.61 (0.44-0.85)	446.14 (52.88-3764)
Nataraj, 2016	Xpert	Culture	0.86 (0.67-0.96)	0.98 (0.94-1.00)	39.71 (12.8-122.9)	0.15 (0.06-0.36)	272.00 (57.24-1293)
Penata, 2016	Xpert	Culture	1.00 (0.16-1.00)	1.00 (0.92-1.00)	36.00 (4.77-271.5)	0.26 (0.05-1.39)	141.00 (6.97-2853)
Suzana, 2016	Xpert	Culture	0.57 (0.18-0.90)	0.91 (0.79-0.98)	6.57 (2.11-20.45)	0.47 (0.20-1.11)	14.00 (2.28-85.94)
Yuan, 2016	Xpert	Composite	0.31 (0.19-0.45)	1.00 (0.63-1.00)	3.16 (0.47-21.08)	0.76 (0.58-1.00)	4.15 (0.49-35.31)
Che, 2017	Xpert	Composite	0.20 (0.11-0.32)	1.00 (0.81-1.00)	4.19 (0.58-30.09)	0.83 (0.71-0.98)	5.04 (0.62-41.24)
Jing, 2017	Xpert	Culture	0.36 (0.20-0.55)	0.96 (0.89-0.99)	8.27 (2.87-23.86)	0.67 (0.51-0.86)	12.43 (3.64-42.43)
Li, 2017	Xpert	Culture	0.40 (0.21-0.61)	0.91 (0.86-0.94)	4.36 (2.27-8.35)	0.66 (0.48-0.91)	6.59 (2.59-16.80)
Pandey, 2017	Xpert	Culture	0.60 (0.15-0.95)	1.00 (0.80-1.00)	10.86 (1.45-81.25)	0.45 (0.19-1.07)	24.00 (1.95-295.1)
Saeed, 2017	Xpert	Culture	0.91 (0.76-0.98)	1.00 (0.97-1.00)	112.49 (15.9-795.3)	0.12 (0.05-0.29)	976.50 (105.4-9047)
Bankar, 2018	Xpert	Culture	0.64 (0.31-0.89)	0.92 (0.88-0.95)	8.11 (4.47-14.69)	0.39 (0.18-0.86)	20.54 (5.60-75.39)
Khan, 2018	Xpert	Culture	0.58 (0.43-0.71)	1.00 (0.98-1.00)	119.98 (16.7-859.2)	0.43 (0.31-0.58)	280.35 (36.55-2150)
Perez-Risco, 2018	Ultra	Culture	0.48 (0.26-0.70)	1.00 (0.29-1.00)	2.39 (0.39-14.53)	0.65 (0.36-1.17)	3.67 (0.35-38.03)
Prakash, 2018	Xpert	Culture	0.67 (0.22-0.96)	0.98 (0.95-0.99)	31.17 (10.14-95.8)	0.34 (0.11-1.06)	91.50 (12.82-653)

Rakotoarivelo, 2018	Xpert	Culture	0.64 (0.31-0.89)	1.00 (0.89-1.00)	20.92 (2.89-151.3)	0.40 (0.20-0.79)	52.80 (5.39-517.1)
		Composite	0.24 (0.10-0.44)	1.00 (0.77-1.00)	4.13 (0.56-30.19)	0.79 (0.62-1.01)	5.22 (0.59-46.07)
Sharma, 2018	Xpert	Composite	0.33 (0.20-0.48)	1.00 (0.88-1.00)	10.88 (1.52-77.81)	0.68 (0.55-0.84)	15.97 (2.00-127.3)
Allahyartorkaman, 2019	Xpert	Culture	0.64 (0.31-0.89)	0.97 (0.94-0.99)	24.98 (8.61-72.50)	0.37 (0.17-0.82)	66.94 (13.8-324.8)
El-Din, 2019	Xpert	Composite	0.02 (0.00-0.12)	1.00 (0.74-1.00)	0.58 (0.06-5.97)	1.03 (0.88-1.21)	0.57 (0.05-6.74)
Kumari, 2019	Xpert	Composite	0.22 (0.11-0.37)	1.00 (0.90-1.00)	8.89 (1.20-65.84)	0.79 (0.67-0.93)	11.31 (1.39-92.13)
Liang, 2019	Xpert	Composite	0.14 (0.09-0.21)	1.00 (0.94-1.00)	9.67 (1.33-70.12)	0.87 (0.81-0.93)	11.16 (1.47-84.43)
Luo, 2019	Xpert	Composite	0.33 (0.27-0.39)	1.00 (0.98-1.00)	63.40 (8.90-451.5)	0.67 (0.61-0.73)	94.41 (12.99-686)
Mechal, 2019	Xpert	Culture	1.00 (0.16-1.00)	0.97 (0.89-1.00)	16.25 (4.70-56.24)	0.26 (0.05-1.43)	62.00 (4.88-788.1)
Meldau, 2019	Xpert	Composite	0.29 (0.17-0.43)	0.99 (0.94-1.00)	24.00 (3.25-176.9)	0.72 (0.60-0.86)	33.20 (4.20-262.3)
	Ultra	Composite	0.38 (0.24-0.53)	0.99 (0.94-1.00)	31.50 (4.34-228.6)	0.63 (0.51-0.79)	49.80 (6.37-389.4)
Tadesse, 2019	Xpert	Culture	0.69 (0.39-0.91)	0.96 (0.89-0.99)	16.27 (5.84-45.35)	0.32 (0.14-0.73)	50.63 (10.79-237)
		Composite	0.30 (0.17-0.46)	1.00 (0.94-1.00)	20.53 (2.80-150.7)	0.70 (0.57-0.85)	29.35 (3.69-233.4)
Tahseen, 2019	Xpert	Culture	0.13 (0.06-0.22)	0.91 (0.86-0.95)	1.41 (0.68-2.91)	0.96 (0.87-1.06)	1.47 (0.64-3.33)
Wang, 2019	Xpert	Culture	0.49 (0.36-0.63)	0.88 (0.78-0.94)	4.04 (2.07-7.87)	0.58 (0.44-0.76)	6.97 (2.92-16.63)
		Composite	0.34 (0.25-0.44)	1.00 (0.85-1.00)	8.64 (1.24-59.95)	0.68 (0.58-0.80)	12.67 (1.65-97.28)
	Ultra	Culture	0.84 (0.72-0.93)	0.74 (0.63-0.84)	3.28 (2.19-4.91)	0.21 (0.11-0.39)	15.44 (6.39-37.32)
		Composite	0.61 (0.51-0.70)	0.96 (0.78-1.00)	14.06 (2.05-96.14)	0.41 (0.32-0.52)	34.57 (4.49-266.1)
Wu, 2019	Xpert	Culture	0.54 (0.33-0.74)	0.90 (0.82-0.95)	5.17 (2.65-10.10)	0.51 (0.33-0.79)	10.10 (3.65-27.93)
		Composite	0.21 (0.14-0.30)	0.95 (0.75-1.00)	4.22 (0.60-29.50)	0.83 (0.72-0.96)	5.08 (0.65-39.98)
	Ultra	Culture	0.74 (0.52-0.90)	0.68 (0.58-0.77)	2.33 (1.61-3.38)	0.38 (0.19-0.77)	6.10 (2.20-16.87)
		Composite	0.45 (0.35-0.55)	0.90 (0.68-0.99)	4.49 (1.18-16.99)	0.61 (0.49-0.77)	7.32 (1.62-33.14)
Zhou, 2019	Xpert	Composite	0.50 (0.12-0.88)	1.00 (0.48-1.00)	3.50 (0.50-24.41)	0.58 (0.27-1.24)	6.00 (0.48-75.34)
Abdelfattah, 2020	Xpert	Culture	1.00 (0.03-1.00)	1.00 (0.48-1.00)	4.67 (0.64-33.91)	0.39 (0.08-1.98)	12.00 (0.49-294.6)
Chen, 2020	Xpert	Composite	0.40 (0.05-0.85)	1.00 (0.16-1.00)	1.71 (0.26-11.47)	0.76 (0.32-1.79)	2.25 (0.15-33.93)
Dahiya, 2020	Xpert	Composite	0.39 (0.20-0.61)	1.00 (0.82-1.00)	8.40 (1.17-60.36)	0.63 (0.45-0.88)	13.33 (1.53-115.8)

Han, 2020	Xpert	Culture	0.27 (0.22-0.34)	1.00 (0.92-1.00)	12.12 (1.73-85.14)	0.74 (0.68-0.81)	16.36 (2.20-121.3)
Hoel, 2020	Ultra	Culture	0.00 (0.00-0.98)	1.00 (0.80-1.00)	6.33 (0.53-76.39)	0.70 (0.31-1.58)	9.00 (0.39-206.5)
Li, 2020	Xpert	Composite	0.15 (0.03-0.38)	1.00 (0.94-1.00)	11.64 (1.37-98.62)	0.83 (0.68-1.01)	14.00 (1.47-133.2)
Sasikumar, 2020	Xpert	Composite	0.71 (0.52-0.86)	1.00 (0.69-1.00)	8.36 (1.26-55.36)	0.33 (0.19-0.57)	25.30 (2.87-223.3)
Sumalani, 2020	Xpert	Composite	0.11 (0.05-0.19)	1.00 (0.94-1.00)	7.67 (1.01-58.46)	0.90 (0.83-0.97)	8.55 (1.07-68.60)
Wang, 2020	Xpert	Culture	0.51 (0.37-0.65)	0.95 (0.91-0.97)	9.28 (5.15-16.72)	0.52 (0.40-0.68)	17.87 (8.28-38.57)
		Composite	0.19 (0.14-0.25)	0.99 (0.94-1.00)	16.15 (2.26-115.6)	0.82 (0.76-0.88)	19.76 (2.67-146.3)
	Ultra	Culture	0.84 (0.71-0.92)	0.80 (0.75-0.85)	4.22 (3.18-5.59)	0.20 (0.11-0.37)	20.66 (9.45-45.19)
		Composite	0.44 (0.37-0.51)	0.99 (0.94-1.00)	37.15 (5.26-262.2)	0.56 (0.50-0.64)	65.83 (8.99-481.9)
Yang, 2020	Xpert	Composite	0.39 (0.30-0.48)	1.00 (0.98-1.00)	64.26 (8.99-459.2)	0.62 (0.54-0.71)	104.21 (14.12-769)
Yu, 2020	Xpert	Composite	0.19 (0.09-0.31)	1.00 (0.87-1.00)	5.50 (0.75-40.49)	0.83 (0.72-0.97)	6.60 (0.81-54.00)
Elbrolosy, 2021	Xpert	Culture	0.78 (0.40-0.97)	0.87 (0.60-0.98)	5.83 (1.53-22.20)	0.26 (0.07-0.88)	22.75 (2.61-198.2)
Gao, 2021	Xpert	Culture	0.16 (0.03-0.40)	1.00 (0.92-1.00)	8.38 (1.00-70.44)	0.83 (0.67-1.02)	10.12 (1.05-97.16)
		Composite	0.11 (0.02-0.29)	1.00 (0.90-1.00)	4.97 (0.59-42.04)	0.89 (0.76-1.04)	5.60 (0.59-53.16)
	Ultra	Culture	0.58 (0.34-0.80)	1.00 (0.92-1.00)	25.14 (3.50-180.8)	0.44 (0.27-0.72)	57.33 (6.59-498.5)
		Composite	0.48 (0.29-0.68)	1.00 (0.90-1.00)	17.38 (2.43-124.5)	0.53 (0.37-0.76)	32.67 (3.93-271.3)
Guo, 2021	Xpert	Composite	0.12 (0.06-0.19)	1.00 (0.85-1.00)	3.07 (0.42-22.36)	0.91 (0.82-1.02)	3.35 (0.42-26.93)
Kim, 2021	Xpert	Culture	0.55 (0.23-0.83)	1.00 (0.96-1.00)	54.92 (7.33-411.6)	0.47 (0.26-0.84)	117.83 (12.40-1119)
Kobra, 2021	Xpert	Culture	1.00 (0.81-1.00)	0.95 (0.82-0.99)	12.35 (4.14-36.80)	0.05 (0.01-0.37)	228.00 (22.17-2344)
Koumeka, 2021	Xpert	Composite	0.42 (0.20-0.67)	1.00 (0.87-1.00)	12.00 (1.65-87.52)	0.59 (0.41-0.86)	20.25 (2.30-178.2)
Lopez-Roa, 2021	Ultra	Culture	0.50 (0.12-0.88)	1.00 (0.97-1.00)	57.00 (7.19-452.1)	0.50 (0.25-1.01)	113.00 (10.17-1255)
Mekkaoui, 2021	Ultra	Culture	0.67 (0.30-0.93)	0.99 (0.92-1.00)	45.33 (6.14-334.8)	0.34 (0.13-0.85)	134.00 (12.01-1495)
Penata-Bedoya, 2021	Ultra	Culture	1.00 (0.29-1.00)	0.93 (0.86-0.98)	10.51 (4.56-24.26)	0.22 (0.04-1.25)	48.57 (4.76-495.7)
Sun, 2021	Xpert	Composite	0.14 (0.09-0.19)	1.00 (0.86-1.00)	3.68 (0.52-25.87)	0.89 (0.81-0.98)	4.12 (0.54-31.56)
