# Supplementary material for: Xpert MTB/RIF Ultra versus Xpert MTB/RIF for diagnosis of tuberculous pleural effusion: A systematic review and comparative meta-analysis
Source: PLoS One. 2022 Jul 11;17(7):e0268483. doi: 10.1371/journal.pone.0268483 (PMC9273090; doi:10.1371/journal.pone.0268483)
Supplement: S3 Table — (PDF) [file pone.0268483.s004.pdf]

S3 Table. Evaluation of factors affecting individual summary diagnostic accuracy estimates from studies on pleural fluid Xpert MTB/RIF assay.

**Factors and categories**		**Mycobacterial culture as reference standard**					**Composite reference standard**				
		**No.**	**Sensitivity**	**I^2**	**Specificity**	**I^2**	**No.**	**Sensitivity**	**I^2**	**Specificity**	**I^2**
Overall		45	0.52 (0.43-0.60)	82.1%	0.99 (0.97-0.99)	85.1%	35	0.21 (0.17-0.26)	81.5%	1.00 (0.99-1.00)	37.6%
Design of study	- Prospective	35	0.55 (0.45-0.64)	85.3%	0.98 (0.96-0.99)	82.5%	26	0.23 (0.18-0.29)	82.5%	1.00 (0.99-1.00)	07.8%
	- Not prospective	10	0.41 (0.27-0.57)	56.3%	0.99 (0.98-1.00)	83.8%	9	0.17 (0.12-0.25)	70.6%	1.00 (1.00-1.00)	0.00%
Case control study	- No	41	0.53 (0.43-0.62)	83.8%	0.99 (0.97-0.99)	86.9%	33	0.21 (0.17-0.26)	82.3%	1.00 (0.99-1.00)	52.3%
	- Yes	4	0.42 (0.24-0.63)	53.1%	0.97 (0.88-0.99)	0.00%	2	Not estimable	-	Not estimable	-
Burden of TB in country of study	- Not high	24	0.57 (0.40-0.72)	65.2%	0.99 (0.98-1.00)	78.2%	9	0.13 (0.06-0.26)	84.8%	1.00 (0.75-1.00)	32.5%
	- High	21	0.48 (0.38-0.58)	85.4%	0.97 (0.95-0.98)	79.2%	26	0.24 (0.19-0.29)	81.0%	1.00 (0.99-1.00)	26.3%
Proportion of TB patients in study	- >50%	4	Not estimable	-	Not estimable	-	22	0.22 (0.16-0.29)	84.6%	1.00 (0.99-1.00)	25.5%
	- <=50%	41	0.55 (0.45-0.64)	78.8%	0.99 (0.97-0.99)	87.4%	13	0.21 (0.15-0.29)	73.9%	1.00 (0.99-1.00)	42.8%
Total sample size	- <=100 patients	22	0.58 (0.38-0.76)	71.0%	0.99 (0.96-1.00)	57.5%	21	0.20 (0.14-0.29)	99.2%	1.00 (0.99-1.00)	0.00%
	- >100 patients	23	0.49 (0.40-0.58)	85.7%	0.98 (0.97-0.99)	90.1%	14	0.22 (0.17-0.28)	84.9%	1.00 (0.99-1.00)	65.1%
Effusion characteristics	- Only exudates	6	0.36 (0.25-0.49)	78.3%	0.98 (0.90-1.00)	27.8%	13	0.20 (0.15-0.25)	79.5%	1.00 (0.98-1.00)	0.00%
	- Transudates also*	39	0.55 (0.45-0.64)	79.2%	0.99 (0.97-0.99)	87.9%	22	0.22 (0.16-0.30)	82.5%	1.00 (0.75-1.00)	76.8%
Nature of sample	- Cryopreserved	4	0.39 (0.27-0.53)	17.1%	0.93 (0.84-0.97)	0.00%	5	Not estimable	-	Not estimable	-
	- Fresh*	41	0.53 (0.44-0.63)	84.0%	0.99 (0.98-0.99)	86.9%	30	0.22 (0.17-0.27)	81.2%	1.00 (0.99-1.00)	41.3%
Fluid centrifugation prior to assay	- Yes	22	0.53 (0.38-0.68)	83.0%	0.99 (0.97-0.99)	91.1%	15	0.20 (0.15-0.27)	80.4%	1.00 (0.98-1.00)	63.8%
	- No*	23	0.51 (0.42-0.61)	82.3%	0.99 (0.97-0.99)	75.6%	20	0.22 (0.16-0.30)	83.2%	1.00 (0.99-1.00)	0.00%

Figures in parentheses are 95% confidence intervals for the summary sensitivity and specificity estimates

I^2 Higgin's inconsistency index, TB Tuberculosis

* Also includes studies where no specific information was available
