# Supplementary material for: Xpert MTB/RIF Ultra versus Xpert MTB/RIF for diagnosis of tuberculous pleural effusion: A systematic review and comparative meta-analysis
Source: PLoS One. 2022 Jul 11;17(7):e0268483. doi: 10.1371/journal.pone.0268483 (PMC9273090; doi:10.1371/journal.pone.0268483)

S2 Fig. Forest plots of studies evaluating sensitivity and specificity of pleural fluid Xpert MTB/RIF assay in diagnosing tuberculous pleural effusion. Solid squares indicate individual study estimates, and horizontal lines represent corresponding 95% confidence limits.

Author, year, and reference standard used in the study	TP	FN	TN	FP	Sensitivity (95% CI)	Specificity (95% CI)
Culture as reference standard						
- Abdelfattah, 2020	1	0	5	0	1.00 (0.03-1.00)	1.00 (0.48-1.00)
- Al-Ateah, 2012	3	0	10	0	1.00 (0.29-1.00)	1.00 (0.69-1.00)
- Causse, 2011	4	0	30	0	1.00 (0.40-1.00)	1.00 (0.88-1.00)
- Hanif, 2011	3	0	8	0	1.00 (0.29-1.00)	1.00 (0.63-1.00)
- Kobra, 2021	18	0	35	2	1.00 (0.81-1.00)	0.95 (0.82-0.99)
- Mechal, 2019	2	0	61	2	1.00 (0.16-1.00)	0.97 (0.89-1.00)
- Penata, 2016	2	0	46	0	1.00 (0.16-1.00)	1.00 (0.92-1.00)
- Saeed, 2017	30	3	125	0	0.91 (0.76-0.98)	1.00 (0.97-1.00)
- Nataraj, 2016	24	4	136	3	0.86 (0.67-0.96)	0.98 (0.94-1.00)
- Elbrolosy, 2021	7	2	13	2	0.78 (0.40-0.97)	0.87 (0.60-0.98)
- Coleman, 2015	9	4	37	0	0.69 (0.39-0.91)	1.00 (0.91-1.00)
- Tadesse, 2019	9	4	90	4	0.69 (0.39-0.91)	0.96 (0.89-0.99)
- Prakash, 2018	4	2	183	4	0.67 (0.22-0.96)	0.98 (0.95-0.99)
- Allahyartorkaman, 2019	7	4	153	4	0.64 (0.31-0.89)	0.97 (0.94-0.99)
- Bankar, 2018	7	4	270	23	0.64 (0.31-0.89)	0.92 (0.88-0.95)
- Rakotoarivelo, 2018	7	4	32	0	0.64 (0.31-0.89)	1.00 (0.89-1.00)
- Pandey, 2017	3	2	17	0	0.60 (0.15-0.95)	1.00 (0.80-1.00)
- Khan, 2018	30	22	207	0	0.58 (0.43-0.71)	1.00 (0.98-1.00)
- Suzana, 2016	4	3	42	4	0.57 (0.18-0.90)	0.91 (0.79-0.98)
- Rufai, 2015	23	19	119	0	0.55 (0.39-0.70)	1.00 (0.97-1.00)
- Kim, 2021	6	5	100	0	0.55 (0.23-0.83)	1.00 (0.96-1.00)
- Wu, 2019	13	11	94	11	0.54 (0.33-0.74)	0.90 (0.82-0.95)
- Wang, 2020	28	27	224	13	0.51 (0.37-0.65)	0.95 (0.91-0.97)
- Vadwai, 2011	5	5	19	0	0.50 (0.19-0.81)	1.00 (0.82-1.00)
- Wang, 2019	28	29	65	9	0.49 (0.36-0.63)	0.88 (0.78-0.94)
- Scott, 2014	59	66	336	21	0.47 (0.38-0.56)	0.94 (0.91-0.96)
- Du, 2015	24	31	70	1	0.44 (0.30-0.58)	0.99 (0.92-1.00)
- Armand, 2011	3	4	1	0	0.43 (0.10-0.82)	1.00 (0.03-1.00)
- Sharma, 2014	37	54	265	8	0.41 (0.30-0.51)	0.97 (0.94-0.99)
- Li, 2017	10	15	178	18	0.40 (0.21-0.61)	0.91 (0.86-0.94)
- Ozkutuk, 2014	2	3	227	0	0.40 (0.05-0.85)	1.00 (0.98-1.00)
- Porcel, 2013	2	3	58	3	0.40 (0.05-0.85)	0.95 (0.86-0.99)
- Mazzola, 2016	8	13	693	0	0.38 (0.18-0.62)	1.00 (0.99-1.00)
- Jing, 2017	12	21	87	4	0.36 (0.20-0.55)	0.96 (0.89-0.99)
- Tortoli, 2012	5	10	312	3	0.33 (0.12-0.62)	0.99 (0.97-1.00)
- Han, 2020	61	162	42	0	0.27 (0.22-0.34)	1.00 (0.92-1.00)
- Moure, 2012	7	19	5	0	0.27 (0.12-0.48)	1.00 (0.48-1.00)
- Friedrich, 2011	5	14	6	0	0.26 (0.09-0.51)	1.00 (0.54-1.00)
- Gao, 2021	3	16	42	0	0.16 (0.03-0.40)	1.00 (0.92-1.00)
- Kim, 2015	2	11	372	3	0.15 (0.02-0.45)	0.99 (0.98-1.00)
- Tahseen, 2019	10	69	182	18	0.13 (0.06-0.22)	0.91 (0.86-0.95)
- Malbruny, 2011	0	2	10	0	0.00 (0.00-0.84)	1.00 (0.69-1.00)
- Safianowska, 2012	0	2	30	0	0.00 (0.00-0.84)	1.00 (0.88-1.00)
- Zeka, 2011	0	4	52	0	0.00 (0.00-0.60)	1.00 (0.93-1.00)
- Zmak, 2013	0	1	41	0	0.00 (0.00-0.98)	1.00 (0.91-1.00)
Composite reference standard						
- Sasikumar, 2020	22	9	10	0	0.71 (0.52-0.86)	1.00 (0.69-1.00)
- Zhou, 2019	3	3	5	0	0.50 (0.12-0.88)	1.00 (0.48-1.00)
- Tortoli, 2012	8	10	312	0	0.44 (0.22-0.69)	1.00 (0.99-1.00)
- Koumeka, 2021	8	11	26	0	0.42 (0.20-0.67)	1.00 (0.87-1.00)
- Chen, 2020	2	3	2	0	0.40 (0.05-0.85)	1.00 (0.16-1.00)
- Dahiya, 2020	9	14	19	0	0.39 (0.20-0.61)	1.00 (0.82-1.00)
- Yang, 2020	47	75	164	0	0.39 (0.30-0.48)	1.00 (0.98-1.00)
- Wang, 2019	37	71	23	0	0.34 (0.25-0.44)	1.00 (0.85-1.00)
- Sharma, 2018	16	32	30	0	0.33 (0.20-0.48)	1.00 (0.88-1.00)
- Luo, 2019	79	160	189	0	0.33 (0.27-0.39)	1.00 (0.98-1.00)
- Yuan, 2016	17	38	8	0	0.31 (0.19-0.45)	1.00 (0.63-1.00)
- Tadesse, 2019	13	30	64	0	0.30 (0.17-0.46)	1.00 (0.94-1.00)
- Lusiba, 2014	25	62	28	1	0.29 (0.20-0.39)	0.97 (0.82-1.00)
- Meldau, 2019	14	35	83	1	0.29 (0.17-0.43)	0.99 (0.94-1.00)
- Friedrich, 2011	5	15	5	0	0.25 (0.09-0.49)	1.00 (0.48-1.00)
- Rakotoarivelo, 2018	7	22	14	0	0.24 (0.10-0.44)	1.00 (0.77-1.00)
- Meldau, 2014	9	31	47	1	0.23 (0.11-0.38)	0.98 (0.89-1.00)
- Kumari, 2019	10	35	36	0	0.22 (0.11-0.37)	1.00 (0.90-1.00)
- Wu, 2019	23	86	19	1	0.21 (0.14-0.30)	0.95 (0.75-1.00)
- Che, 2017	12	48	18	0	0.20 (0.11-0.32)	1.00 (0.81-1.00)
- Wang, 2020	40	168	83	1	0.19 (0.14-0.25)	0.99 (0.94-1.00)
- Yu, 2020	10	44	26	0	0.19 (0.09-0.31)	1.00 (0.87-1.00)
- Porcel, 2013	5	28	34	0	0.15 (0.05-0.32)	1.00 (0.90-1.00)
- Li, 2020	3	17	62	0	0.15 (0.03-0.38)	1.00 (0.94-1.00)
- Liang, 2019	22	133	64	0	0.14 (0.09-0.21)	1.00 (0.94-1.00)
- Sun, 2021	29	181	24	0	0.14 (0.09-0.19)	1.00 (0.86-1.00)
- Christopher, 2013	4	26	61	0	0.13 (0.04-0.31)	1.00 (0.94-1.00)
- Guo, 2021	12	92	23	0	0.12 (0.06-0.19)	1.00 (0.85-1.00)
- Gao, 2021	3	24	34	0	0.11 (0.02-0.29)	1.00 (0.90-1.00)
- Sumalani, 2020	9	75	64	0	0.11 (0.05-0.19)	1.00 (0.94-1.00)
- Kim, 2015	5	44	339	0	0.10 (0.03-0.22)	1.00 (0.99-1.00)
- Trajman, 2014	2	57	26	0	0.03 (0.00-0.12)	1.00 (0.87-1.00)
- El-Din, 2019	1	45	12	0	0.02 (0.00-0.12)	1.00 (0.74-1.00)
- Kim, 2015	0	6	26	0	0.00 (0.00-0.46)	1.00 (0.87-1.00)
- Zeka, 2011	0	8	48	0	0.00 (0.00-0.37)	1.00 (0.93-1.00)

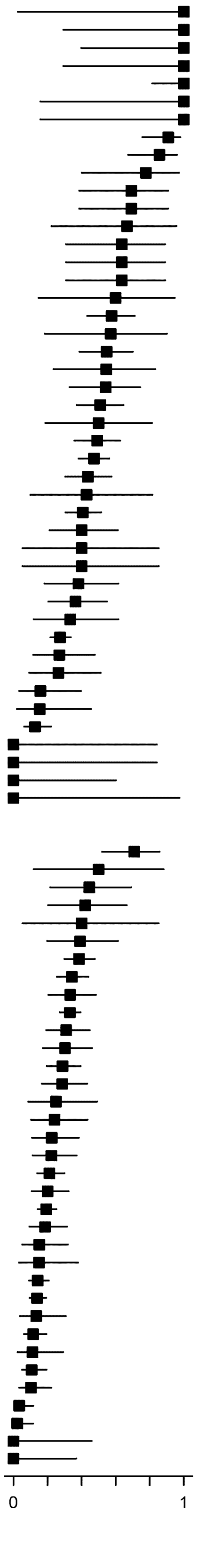

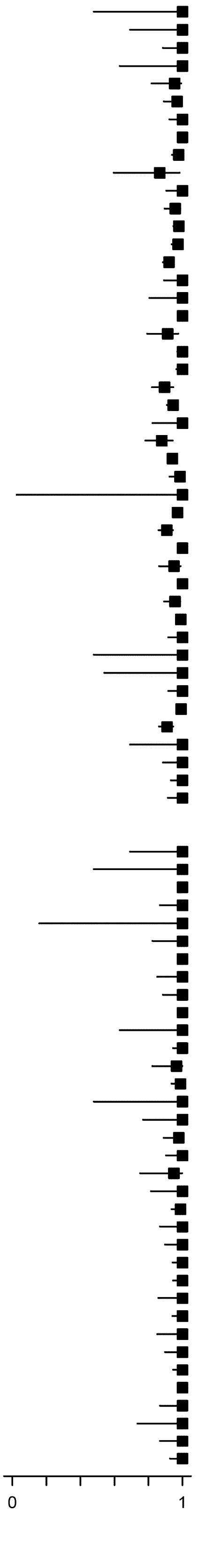

Supplement: S1 Fig — Solid squares indicate individual study estimates, and horizontal lines represent corresponding 95% confidence limits. (PDF) [file pone.0268483.s005.pdf]
