# Supplementary material for: Xpert MTB/RIF Ultra versus Xpert MTB/RIF for diagnosis of tuberculous pleural effusion: A systematic review and comparative meta-analysis
Source: PLoS One. 2022 Jul 11;17(7):e0268483. doi: 10.1371/journal.pone.0268483 (PMC9273090; doi:10.1371/journal.pone.0268483)

S3 Fig. Summary receiver operating characteristic (SROC) curves from bivariate models summarizing diagnostic performance of pleural fluid Xpert MTB/RIF using culture (top left) or composite criteria (top right) as reference standard, and pleural fluid Xpert MTB/RIF Ultra using culture (bottom left) or composite criteria (bottom right) as reference standard. Each individual study on diagnosis of tuberculous pleural effusion is represented by an open circle, whose size is proportional to the inverse standard error of sensitivity and specificity. The square represents the summary estimate of test accuracy, with the surrounding dashed zone outline denoting the 95% confidence region around this estimate. The outer dotted zone represents the 95% prediction region (area within which one is 95% certain the results of a new study will lie).

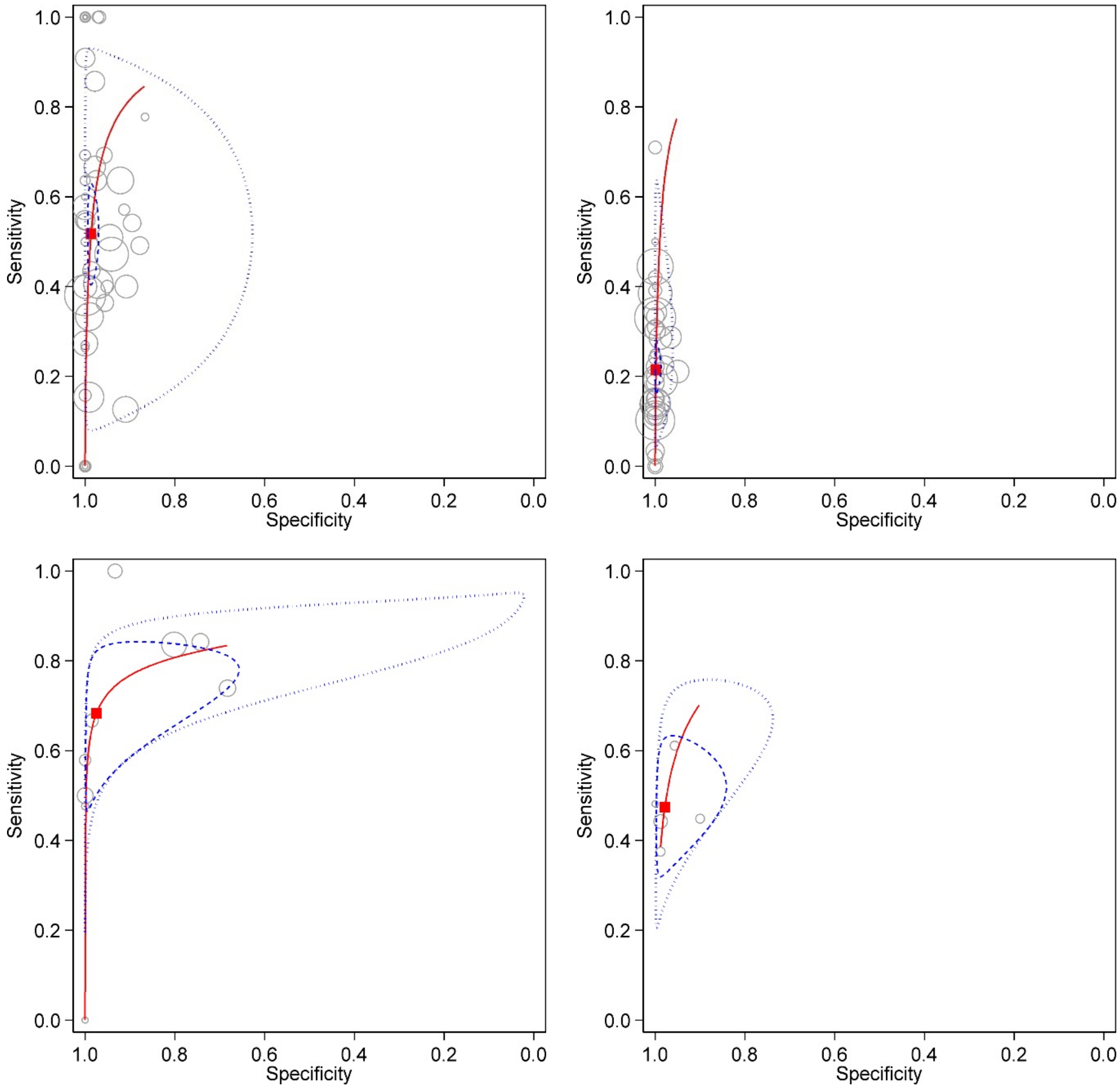

Supplement: S2 Fig — Each individual study on diagnosis of tuberculous pleural effusion is represented by an open circle, whose size is proportional to the inverse standard error of sensitivity and specificity. The square represents the summary estimate of test accuracy, with the surrounding dashed zone outline denoting the 95% confidence region around this estimate. The outer dotted zone represents the 95% prediction region (area within which one is 95% certain the results of a new study will lie). (PDF) [file pone.0268483.s006.pdf]
