# Supplementary material for: Xpert MTB/RIF Ultra versus Xpert MTB/RIF for diagnosis of tuberculous pleural effusion: A systematic review and comparative meta-analysis
Source: PLoS One. 2022 Jul 11;17(7):e0268483. doi: 10.1371/journal.pone.0268483 (PMC9273090; doi:10.1371/journal.pone.0268483)

S4 Fig. Forest plots of studies evaluating sensitivity and specificity of pleural fluid Xpert MTB/RIF Ultra in diagnosing tuberculous pleural effusion. Solid squares indicate individual study estimates, and horizontal lines represent corresponding 95% confidence limits.

Author, year, and reference standard used in the study	TP	FN	TN	FP	Sensitivity (95% CI)	Specificity (95% CI)
Culture as reference standard						
- Penata-Bedoya, 2021	3	0	84	6	1.00 (0.29-1.00)	0.93 (0.86-0.98)
- Wang, 2019	48	9	55	19	0.84 (0.72-0.93)	0.74 (0.63-0.84)
- Wang, 2020	46	9	190	47	0.84 (0.71-0.92)	0.80 (0.75-0.85)
- Wu, 2019	17	6	71	33	0.74 (0.52-0.90)	0.68 (0.58-0.77)
- Mekkaoui, 2021	6	3	67	1	0.67 (0.30-0.93)	0.99 (0.92-1.00)
- Gao, 2021	11	8	42	0	0.58 (0.34-0.80)	1.00 (0.92-1.00)
- Lopez-Roa, 2021	3	3	112	0	0.50 (0.12-0.88)	1.00 (0.97-1.00)
- Perez-Risco, 2018	10	11	3	0	0.48 (0.26-0.70)	1.00 (0.29-1.00)
- Hoel, 2020	0	1	17	0	0.00 (0.00-0.98)	1.00 (0.80-1.00)
Composite reference standard						
- Wang, 2019	66	42	22	1	0.61 (0.51-0.70)	0.96 (0.78-1.00)
- Gao, 2021	13	14	34	0	0.48 (0.29-0.68)	1.00 (0.90-1.00)
- Wu, 2019	48	59	18	2	0.45 (0.35-0.55)	0.90 (0.68-0.99)
- Wang, 2020	92	116	83	1	0.44 (0.37-0.51)	0.99 (0.94-1.00)
- Meldau, 2019	18	30	83	1	0.38 (0.24-0.53)	0.99 (0.94-1.00)

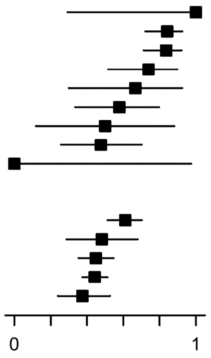

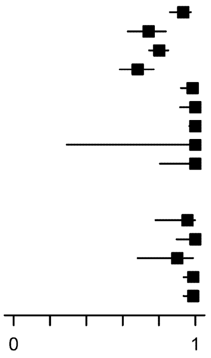

Supplement: S3 Fig — Solid squares indicate individual study estimates, and horizontal lines represent corresponding 95% confidence limits. (PDF) [file pone.0268483.s007.pdf]
