# Supplementary material for: Xpert MTB/RIF Ultra versus Xpert MTB/RIF for diagnosis of tuberculous pleural effusion: A systematic review and comparative meta-analysis
Source: PLoS One. 2022 Jul 11;17(7):e0268483. doi: 10.1371/journal.pone.0268483 (PMC9273090; doi:10.1371/journal.pone.0268483)
Supplement: Attachment — Submitted filename: Answer to reviewer comments.docx [file pone.0268483.s008.docx]

**Responses to comments from Reviewer #1:**

Comment:

Alternatively, the authors could state more clearly that, in those with a culture-negative TPE, the positive PCR tests correlated better with a good TB treatment outcome than would be expected by chance. Papers reporting such data would require absence of a blood neutrophilia and follow-up for 1-5 years after treatment completion (untreated TPE results in post-primary TB usually within 5 years).

Response:

We had focussed sorely on diagnsotic accuracy information and not on treatment oucomes. No study provided detailed information on treatment oucomes stratified by culture or PCR results, or by pleural fluid characterestics. This has been added as a limitation in Discussion (para 3, lines 365-366).

Comment:

The abstract should report the I-squared value, indicating that most of the differences were due to heterogeneity in the studies.

Response:

We have provided the I-squared values in abstract (lines 76-81).

Comment:

The introduction should note the prevalence of IS1081 in mycobacterial species other then Mtb sensu stricto. This will provide a background for discussion of the lower specificity of Xpert Ultra (M bovis, BCG, M smegmatis etc., and also related to the restriction enzyme used).

Response:

Both IS6110 and IS1081 sequences are present in in M. tuberculosis complex, but not in other mycobacteria. This has now been mentioned in Introduction (para 2, lines 114-115).

Comment:

The methods should indicate the nature of the composite score (see above comments). Were different criteria weighted or given equal scores? Line 364 implies that they had data related to the different composite scores.

Response:

A variable and wide range of clinical, laboratory and outcome parameters were used in varying combinations to define the composite reference standards, and it was not possible to characterize or summarize this information further. We highlighted this in Discussion (para 3, lines 365-366)

Comment:

The conclusion should include the “more information” required to better evaluate the Ultra test (also noted above in the 2nd and 3rd paragraphs).

Response:

As suggested, we have added text to the last line in Conclusion (lines 386-387).

Comment:

The supplemental figure 1 (risk of bias…) is of such significance that it should be included in the main text.

Response:

We have now included the figure on risk of bias and applicability concerns summary as Fig 2 in the main manuscript, and renumbered the subsequent figures in main manuscript and online supplement accordingly.

Comment:

Typographical errors. Line 116: Health not health

Response:

We apologise for this inadvertant error, and the same has now been corrected (line 118).

Responses to comments from Reviewer #2:

Comment:

In lines 321 and 326, about the summary of findings, the text refers to table three, but these data are showed in table two; can the authors check?

Response:

We apologise for this typographical error related to citation for Table 2, and the same has now been corrected in Results (last para, lines 319, 322, 327).

Comment:

In figure 1, the workflow of analysis display that the authors used 74 publications for data synthesis, but in lines 229 and 253, the authors state the use of 45 publication with Mtb culture as the reference standard and 35 publications with composite reference standard respectively. The same numbers, 45 and 35, are also reported in supplemental table three. Can the authors better specify if they used 74 or 80 publications for the analysis?

Response:

There were a few studies that provided data for both compositie and culture-based reference standards, and hence the overlap in numbers. This has already been clarified while reporting the study characterestics (Results, para 1, lines 218-220).
